# All that was missing from the students of the University of Patras and the return to a new daily life after quarantine

**DOI:** 10.1192/j.eurpsy.2022.1346

**Published:** 2022-09-01

**Authors:** A. Katsifara, K. Argyropoulos, D. Avramidis, E. Jelastopulu

**Affiliations:** School of Medicine, University of Patras, Public Health, Rio, Greece

**Keywords:** UNIVERSITY, Covid-19, students, quarantine

## Abstract

**Introduction:**

The pandemic that broke out by the new coronavirus SARS-CoV-2 and the imposition of restrictive measures to reduce the dispersion, affects both the physical and mental health of all population groups.

**Objectives:**

The main objective of the study was to investigate how these measures have impacted the students during the first quarantine period (Spring 2020). Also we wanted to know what they lacked most after the six-weeks-lockdown.

**Methods:**

More than 2,000 students from all Schools of the University of Patras participated in the research, completing an online questionnaire. Emphasis was placed on the question “What is the FIRST thing you will do immediately after lifting the measures”. The open last option ‘Other’ was qualitative investigated with thematic analysis by gender.

**Results:**

The answer options of the evaluated question were to ‘Go out for coffee/food/drink/fun with friends’ (58%) or ‘with family’ (5%), to ‘Visit beauty and hair salons’ (16%), to ‘Travel’ (6%), or to ‘Go shopping’ (2%). The option ‘Other’ was answered by 246 (13%) students. The thematic analysis revealed 13 categories, with first place ‘Restoring immediately social life without restrictions’, followed by ‘Seeing and being together with boyfriend/girlfriend’, but at the same time ‘Continue to be careful and take self-restraining measures after the end of the quarantine’.

**Conclusions:**

Students of both genders lacked mainly social life and companionship. The need to return to a new daily routine with protection measures that limit both exposure to the new virus and the spontaneity, is obvious.

**Disclosure:**

No significant relationships.

